# A Transdisciplinary Framework to Unlock the Potential Benefits of Green Spaces for Urban Communities Under Changing Contexts

**DOI:** 10.1093/biosci/biad009

**Published:** 2023-03-17

**Authors:** Brenda B Lin, Erik Andersson

**Affiliations:** CSIRO Land and Water, Brisbane, Queensland, Australia; Stockholm Resilience Centre, Stockholm University, Stockholm, Sweden; Ecosystems and Environment Research Program, University of Helsinki, Helsinki, Finland; Research Unit for Environmental Sciences and Management, North-West University, in Vanderbijlpark, South Africa

**Keywords:** socioecological systems, integration science, interdisciplinary processes, urban studies, multifunctional systems

## Abstract

New urban models increasingly seek to create more sustainable, livable, and healthier cities by reinvigorating green space. In this article, we highlight and briefly review several main but disconnected areas of study in which the factors that frame human–environment interactions and therefore also influence the potential well-being outcomes of those interactions are studied. We then use the intersection of affordance theory and socio-institutional programming to provide a conceptual framework that ties together these spheres of research, and we discuss some critical keys for enabling different positive green space experiences. Urban communities are not homogeneous, and accounting for the intersection between individual differences and landscape programming opens up more diverse pathways for affording positive human–environment interactions and different well-being outcomes.

Urban green spaces offer opportunities for both physical activity and passive uses of green space. Spending time in green space, whether it is moving through a park, having a picnic, or even enjoying the view from a home has been associated with mental health benefits, such as reduced stress, reduced depression, and lower anxiety levels, and increased opportunities for physical activity outdoors can reduce problems with obesity and cardiovascular disease (Richardson et al. 2013, Gascon et al. 2015, Dean et al. 2018, Basu et al. 2019). The ability to provide these benefits also in a socially distanced way became especially important during COVID-19, when many people looked to green spaces for exercise, as well as to spend time out of their homes (Slater et al. 2020, Bowe et al. 2022). Research during COVID-19 also highlighted the importance of having a “green view” of nearby nature during home isolation, because these views were significantly associated with higher life satisfaction and reduced depression and stress (Soga et al. 2021).

With increasing evidence for and recognition of green space contributions to urban attractiveness and livability, local governments worldwide are investing in green spaces with the intention of delivering well-being benefits to diverse communities and diverse needs (Lindholst et al. 2016, Dobson 2021, Garcia-Lamarca et al. 2021). Not only do these spaces provide important cultural benefits to the urban community, but local governments invest in green spaces for the other environmental services that can hugely benefit the community, such as air and water filtration, thermal comfort, and stormwater regulation (Lai et al. 2019, Biswal et al. 2022). For example, new urban models after COVID-19 increasingly seek to create more sustainable, livable, and healthier cities by reinvigorating green space and taking advantage of these numerous benefits (Nieuwenhuijsen 2021). In support of this agenda, many studies have been performed to better understand questions of green space use and measurable benefits on the basis of proximity of nearest green space to neighborhoods or facilities available within these spaces (Kabisch et al. 2016). Often, the idea of a 10-minute walking radius (400–500 meters) has been adopted to consider whether green spaces are in close enough proximity to neighborhoods to be easily available. Proximity has been seen as particularly important, because it is considered a factor in increasing the frequency of visits (Neuvonen et al. 2007). However, equitable access to and benefit from green space is complicated, as is shown by a rich body of diverse studies on the use and accessibility of urban space, providing a critical lens for scrutinizing conventional approaches focused on increasing the quantity and quality of green spaces (e.g., Biernacka and Kronenberg 2019).

Bringing these strands of study and understanding together and using them to carefully plan and promote access to green space use for people with different needs and preferences remains a challenge, despite increasing attention to integration and interdisciplinary studies. We highlight and briefly review several main areas of study that investigate aspects of human–environment interactions in urban green space and health benefit. These areas represent some of the main disciplines that have developed approaches to investigate different aspects of this interaction, which provides a general description of the foundational areas of research. We then provide a conceptual framework that ties together these separate spheres of research and discuss some critical keys for integrating understanding and for unlocking green space potential.

## Landscape architecture and urban planning

Traditionally, green space design has been considered through the lens of landscape and urban planning to understand the spatial distribution of green space across the landscape and to manage them for the provision of benefits and services for urban communities (Ekkel and de Vries 2017, Semeraro et al. 2021). On the basis of this philosophy, many studies have been performed to better understand green space use and the measurable benefits of proximity to nearby green space, as well as the facilities available within these spaces (Kabisch et al. 2016). A large set of research has been concentrated on the accessibility and availability of green spaces across the landscape in order to reduce barriers to use by ensuring the green spaces are near where people live and work and therefore immediately available to use when desired (Biernacka and Kronenberg 2018, Barber et al. 2021). The increasing availability of green spaces includes not just the size of individual green spaces but the provision of multiple different types of greens spaces that may have different purposes—either through larger spaces with different areas or through many smaller spaces that are designed for different uses (Coolen and Meesters 2012, Zhang and Zhou 2018). However, much of the urban planning literature highlights that park distribution and quality can be highly unequal, with low socioeconomic status and ethnic minority communities having access to fewer acres of parks, parks with lower quality, and less maintenance and safety within the parks (Rigolon 2016).

The accessibility of green spaces has also been a significant area of research in this field, with studies developed to understand how different people travel to or move through green spaces (Lee and Hong 2013, Wei 2017). This has been especially studied for certain user groups, such as children or disability advocates, to ensure that parks can be accessed safely and with appropriate pathways and facilities (Perry et al. 2018, Guo et al. 2019). Issues of transport options to parks have been considered to better understand equity of access. For example, one study in Montreal showed that children's access to parks differed on the basis of location, gender, family, and income (Reyes et al. 2014), and another study showed that access to parks that are farther away may be facilitated by improved public transport. Facilities within a park may also affect the accessibility or usability of a park; facilities that provide bathrooms and water fountains may support the use of parks by specific groups of users or neighborhoods (Wu et al. 2017, Pearsall and Eller 2020), and such provision of amenities and infrastructure can increase the inclusivity of park use (Smiley et al. 2016). For example, in a survey of park users in Portland, Oregon, the provision of bathrooms, more garbage and recycling bins, more shade, drinking fountains, and furniture were all highlighted by visitors across the board as facilities that would increase their use of and access to urban green spaces (Talal and Santelmann 2021).

However, the accessibility and availability of green space can only be understood within the urban governance context they are situated in. More or less comprehensive urban planning dovetails with the interests and activities of entities such as private industry, commercial and housing developers, and community groups to determine the quality, quantity, and accessibility of green spaces (Pauleit et al. 2019). Each of these actors has different motivations and behaviors regarding the decision-making frameworks for available land (Anguelovski et al. 2019, Kim et al. 2019). Urban green space on public land can often be supplemented by considered and deliberate green space design on private land; however, the quality of this space can be difficult for local governments to control (Aronson et al. 2017).

## Urban sociology and governance

Studies of health disparities and other inequalities indicate that the means and pathways for achieving optimal health outcomes vary for different groups of people. Rules, laws, policies, and resources often create unequally distributed opportunities to pursue different pathways to positive health outcomes. Environmental justice and health studies have clearly shown how, for example, residential segregation can adversely affect health behaviors, crime, social context, and even access to medical care (Jennings et al. 2019, Williams et al. 2019). Green spaces, especially of high quality, tend to be unequally distributed across cities and are often fenced in or have other permanent or variable access restrictions (opening hours, temporary species, habitat protection, etc.; Matthews et al. 2015, Biernacka and Kronenberg 2018). The ongoing privatization of urban space (Webster, 2002, 2007) can be expected to further limit options for accessing green space benefits (e.g., Wolff et al. 2022). This is especially true for marginalized groups and less resourced neighborhoods, who tend to be both more exposed to environmental hazards and have fewer opportunities to realize desired use of green space, potentially leading to a downward spiral in which the neglect or lacking maintenance of existing green spaces may reduce their attractivity or functionality (e.g., Nassauer and Raskin 2014).

Context, not just biophysical or interpersonal, is decisive for how and when people may use green spaces. Place identity and the legibility of green spaces—and, therefore, their accessibility and attractiveness—are circumscribed by limited understanding of and investment in multiple forms of appropriation and control, active programming or placemaking, and recognition of social embedding and care (e.g., Anguelovski et al. 2022). Institutions; socially constructed and formalized structures; and processes such as policies, property rights, codes of social life, norms, and social control regulate the interaction and expectations among actors and between actors and greenspaces (North 1990, Vatn 2007, Webster 2007, Ostrom 2009). These factors influence who is likely to use which green spaces, how the green spaces are used, and the outcomes of that use in terms of health and well-being. For example, disability studies have explored and described how the social, economic and physical environment cause the loss or a limitation of opportunities for certain groups to take part in life or to make use of “available” resources at an equal level with others (e.g., Burchardt 2004). Social relations also matter, as is the degree to which a potential user is included in the social networks and context around the green spaces. For example, a sense of social inclusion may mitigate low levels of social cohesion in a community and perceived antisocial behavior (Seaman et al. 2010). Being disconnected from social networks or estranged from democratic processes and decision-making may reduce green space use by, for example, discouraging people to visit nearby parks (Berney 2010). Little social context or few interpersonal relationships may lead to people not feeling welcome or feeling that their use of the green space would be at odds with others’.

Beyond the use of existing green spaces and the additional infrastructure that allows for its use (affordable transportation, etc.), groups also vary in their ability to influence the urban landscape over time. Political processes and their openness to participation empower and disempower groups differentially, and the overall governance system decides who can be part of “public” decision-making and what mandate they have.

## Leisure studies and public health

Green spaces have also been studied with the interest of increasing public health through increased opportunities for leisure and recreational activities that contribute to physical and mental health (Mowen et al. 2017, Sugiyama et al. 2018). Although many topics overlap with urban planning, such as increasing the size of green space available and decreasing the distance to access green space, the focus has been frequently directed to the implications that the design of parks have on access to benefits, particularly health benefits (Larson et al. 2016). For example, a study of park use for physical activity in Los Angeles showed that physical activity was correlated with park size; however, parks in high-poverty neighborhoods were underused, regardless of their size, implying other issues of access beyond availability (Park et al. 2018). The design of parks and where they are situated within the built environment can also affect the ability of people to actively access them, which, in turn, affects the walking and cycling activity of communities that may be used to increased physical activity through incidental exercise in daily life (Giles-Corti et al. 2005, Henderson and Bialeschki 2005). Much of the public health literature has highlighted that green space access and use can be highly inequitable, and more must be done to address public green space use to address environmental health disparities in cities (Ward Thompson et al. 2016, Cole et al. 2017).

Parks and recreation departments are becoming increasingly involved in working with health agencies to address the health and wellness of communities through the planning of programs and recreational activities that address issues such as mental health, obesity, and food security (Risisky and MacGregor 2021). Leisure-time physical activities can help prevent chronic disease, and park prescriptions are increasingly considered to promote the use of parks and to help individuals achieve the recommended levels of physical activity in a natural environment (Sallis 2015, PARKRx 2016). However, the promotion and development of programs to help communities get physically active may be required. For example, active programming, such as free physical activity classes offered by kinesiology students at a university in San Fernando, California, in a low-income, predominantly Latino neighborhood park, led to an increase in moderate to vigorous physical exercise for park users and especially for female park users (Han et al. 2015). In another study of outdoor fitness equipment, park users viewed this equipment as potentially beneficial, but many required programs to teach and encourage their use (Copeland et al. 2017). It is also important to note that the environmental quality of a green space must be managed through appropriate lighting and cleanliness to make it a safe and attractive space in which to spend time (Fongar et al. 2019, Yeshitela 2020, Zhang et al. 2021).

## Environmental psychology

Psychological research has shown that frequent interactions with green space are associated with positive measures of health and well-being, with consistent evidence across different populations (Lin et al. 2014, Liu et al. 2017a, Ayala-Azcárraga et al. 2019). In addition, those who live in close proximity to parks and other natural areas experience an improvement to their mental health, well-being, and other life satisfaction traits (Sturm and Cohen 2014). Multiple pathways may provide these benefits. One theory from environmental psychology suggests that immersion of an individual in a natural environment may buffer physiological and emotional stress while promoting restoration and reducing cognitive fatigue, but green spaces also promote social cohesion, a sense of place, and a place to recreate as a way to alleviate stress (Ulrich et al. 1991, Kaplan 1995, Berman et al. 2008, Sugiyama et al. 2008). Because of the benefits that can be achieved, mental health professionals are considering “green prescriptions” and “green doses” to help individuals take advantage of the therapeutic effects of spending time in green space (Van den Berg 2017, Rogerson et al. 2020).

What is less discussed are the psychological attributes of individuals that may influence decisions on green space use or benefits gained. Both time spent in parks and psychological variables have been shown to be relevant predictors of well-being (Scopelliti et al. 2016). Psychological factors such as an individual's affinity, connection, or orientation to nature have been shown to have strong correlations with park visitation, with individuals willing to spend more time in green spaces, as well as to travel a greater distance to visit green spaces (Lin et al. 2014, Shanahan et al. 2015); however, these psychosocial attributes are also often related to the sociodemographic traits, such as higher education levels or high income, often associated with greater nature connection (Lin et al. 2014, Shanahan et al. 2015, Liu et al. 2017b). Those who have a higher salary may be able to live in greener areas or to have more control over how they spend their leisure time, providing more opportunities for green space use, whereas social disadvantage may lead to a lack of time for or knowledge about green space use (Byrne 2012, Shanahan et al. 2015).

Recent research investigating nature connection using a database of twins showed that an individual's affinity for nature is partly genetic (46%), but other factors, such as experience and knowledge, are important in developing a desire to spend time outdoors (Chang et al. 2022). For example, other research has shown that an individual's childhood experiences may affect patterns of green space use and attitudes toward nature as an adult (Thompson et al. 2008, Zhang et al. 2014). A study of university students in Provo, Utah, showed that daily interactions with green space in childhood were associated with more frequent green space use as a university student (Holt et al. 2019). Activities and preferences for green space use may also differ on the basis of cultural experiences with nature (Henderson and Bialeschki 2005). Certain cultures may have a greater fear of dogs because of a different cultural relationship, or minority individuals may be wary of racism in public spaces (Madge 1997). The role of gender may also be highly relevant, with many women choosing when and where they use green space because of a fear of crime or harassment (Sreetheran and Van Den Bosch 2014). Regardless, this is a burgeoning field of research that will help practitioners understand the drivers and barriers of green space use in different contexts.

## Bringing Together Knowledge Areas and Creating a Multidisciplinary Framework of Understanding

While the brief review above covers the different but intersecting fields of study about green space use and their benefits to urban communities, bringing these separate strands of research together to gain an integrated understanding of human–environment interactions will be useful in improving green spaces and encouraging individuals to engage with them.

**Figure 1.**
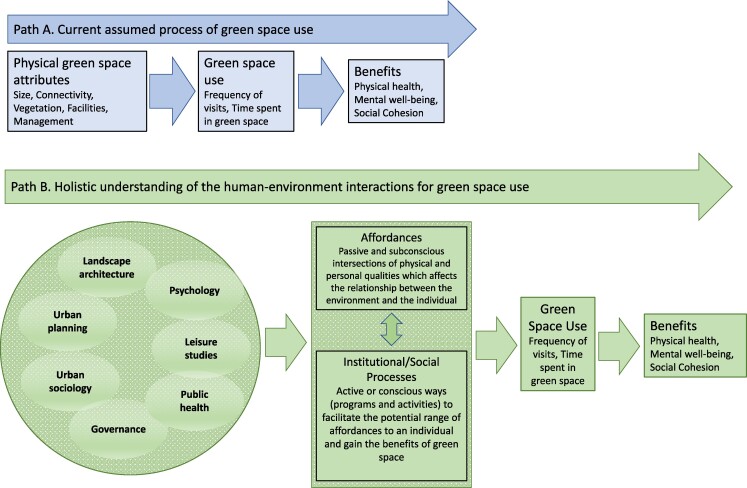
Although the current paradigm primarily follows path A, to develop strategies that increase green space use for health benefits, governments must consider the interaction of multiple areas of research and knowledge in order to design policies that encourage and allow for human–environment interactions that yield multiple benefits (path B). We propose that an understanding of affordances and of how institutional and social systems affect these affordances is necessary to understand and integrate these different spheres of knowledge.

Traditional urban planning processes generally envisage impact as unidirectional (figure 1, path A; Heft 2010). This pathway aims to ensure that there is enough green space and that it is of sufficient quality to attract people into that space in order to deliver the health and well-being benefits it has the potential to offer. However, other research areas highlight the varied and context specific decisions that influence how and when an individual uses green space. Because personal and psychological traits can influence people's use of green spaces, it is necessary to develop a holistic framework that takes into account different spheres of understanding to design green space policy that prioritizes and guides coherent strategies for green space design that addresses community needs ­(figure 1, path B). Although physical green space attributes may provide a number of qualities that attract end users, personal attributes may affect what qualities are desired from the green space.

We propose that, to integrate these spheres, it is important to consider the concept of affordances and the role of institutional and social processes in providing a pathway for integrating different types of knowledge.

### Affordances and green space

*Affordances* is a term coined by Gibson (1979) to describe what an environment “offers the animal, what it provides or furnishes… It implies the complementarity of the animal and the environment” (Gibson 1979, p. 127). The term is used to describe the interactions that could occur between an environment and an animal—in this case, a human—through opportunities or constraints of how a person perceives a particular space. It is a relational term that changes on the basis of context, because the suitability or use of a space can change over time, depending on not only how the space changes but also on how the individual may change. For example, the intentions and capabilities of an individual may change over time, such that a child may consider interacting with their environment differently from how a teenager or an adult would as their physical or cognitive capabilities change (Lennon et al. 2017). Thompson (2007) highlighted that people of various age groups experience parks differently, with children seeking to build dens, teenagers seeking spaces to hang out, young parents wanting places to socialize with each other as they observe their children safely playing, and the elderly preferring traditional flower gardens with benches for rest. It is important to note that the term *affordances* is used in a wide range of fields, including psychology, human–computer interactions, and design and communication, as well as across many other science and technology areas, because it can be used to describe the interaction between humans and other environments beyond the natural environment.

The concept of affordances is focused not on the attributes of either the environment or the animal; rather, it is based on the relationship between the two, and that relationship can be real or perceived (e.g., regardless of the danger of a green space, it may be perceived as unsafe; Chemero 2018). This concept can be especially useful in understanding the interaction between physical green space attributes and personal or community attributes, because it acknowledges that the relationships may be dynamic, and therefore, the choices an individual makes regarding whether and how to use a green space may be highly dependent on their capabilities and on their perceptions of what they are able to do in a green space. Therefore, an individual's attributes will necessarily influence their relationship with each type of green space they encounter (Miller et al. 2014). Heft (2010) provided an example by illustrating that a trail through a wooded area can afford an opportunity for walking, but this may be constrained by other spaces that cut off the trail—in this example, a pond. Other opportunities may become available (e.g., swimming for physical exercise or to cool oneself down, feeding ducks, sitting and fishing), but this will depend greatly on the individual and their experiences as their environment changes.

Affordances in green space help highlight that planning must occur with an understanding of how people with different physical and psychological capacities, interests, and needs relate to different environments, and this is especially important if health and well-being benefits are to be gained within these spaces (Lennon et al. 2017). The design and configuration of green space must be considered through the potential relationships that people perceive of or will have with them. Although one group of users will desire green spaces for recreational purposes, another group will want solitude and to spend time observing nature. These two separate types of use may not be easily combined in one space, and they may serve two different populations or potentially the same population at different times of their day or life. The perceptions of park safety may change depending on the time of day that a park is used, highlighting that relationships between end users and green space design will have to be considered across a day. Considering the network of green spaces available to the wide range of users who would like to use the green space in different ways dependent on context can help reduce the potential constraints perceived by end users while providing diverse opportunities (Haaland and Van Den Bosch 2015).

### Institutional and social processes to support green space use

Although affordances help to highlight the relational value between green spaces and urban residents, they also highlight that these relationships change depending on how the environment or the individual changes. Institutional and social processes are a tangible means of possibly affecting peoples’ perceived affordances of green space; they can engage individuals to reduce the constraints of green space use while providing new opportunities.

Institutional and social processes can include a range of actions, such as providing programs or activities in parks to help support and teach people different ways of interacting with the park. These programs and activities may include a large number of options, including festivals and events hosted in urban green spaces, sports and health programs, and educational activities for the community. All such programs can help provide opportunities for people to learn alternative ways to use a green space and provide an opportunity to interact with a space that may have been previously unknown to them. Specifically, increasing affordances may help an array of users by encouraging them to actualize the affordance, help an individual gain confidence to use that green space, encourage greater frequency and range of uses of that green space, and enhance the potential of that green space to provide health and well-being benefits (Withagen et al. 2012).

For example, in a study of inner-city, low-income seniors in Los Angeles, focus groups within this community indicated that they faced many impediments to using green spaces. What they desired was a set of programs that created opportunities for socializing, increased safety and security within the parks and along access routes, greater opportunities for senior-specific exercise, and aesthetic and natural elements for increased nature connection (Loukaitou-Sideris et al. 2016). In another example of activation to encourage green space use, the installation of equipment, such as fitness zones (exercise precincts) in parks, led to an increase in new users coming to the park and had a greater impact in high-density areas with limited resources (Cohen et al. 2012). However, research has also shown that marketing and outreach are required to communicate programs and activation of green spaces in order to bring people to these spaces (Derose et al. 2014).

Social or active programming can also be beneficial by providing residents with a way to connect to the community and to create a sense of ownership or place making. Enhanced social activities may increase social affordances that provide an individual with the agency to change and adapt the space in order to increase opportunities for use or to reduce specific constraints (Rasidi et al. 2012). For example, community gardens integrated into urban green spaces provide opportunities for a different use of the space, as well as a place for social capital and the integration of different groups within the community. Specific programming within gardens can help members of the community learn more about nature, interact corporeally with plants and soil, and understand processes such as food production (Lin et al. 2018). However, there are additional issues with the perceptions of community gardens as private green space if the ownership and governance of the space are not clear (Hou and Grohmann 2018). Perceptions about the ownership of a space is highly relevant, because questions of ownership severely limit the affordances of those around that particular space (Macintyre et al. 2019).

Because affordances are temporally and spatially dynamic, creating programs that target different age groups, neighborhoods, or types of parks is important to reach a wide range of users. By helping individuals understand how to interact with green spaces through programs and other types of activation, increasing potential affordances can also provide individuals with a greater ecological agency to spend time within these spaces (Withagen et al. 2012).

## Unlocking the Potential of Green Spaces

There is a growing recognition that, to truly understand how urban green spaces are used, an interdisciplinary set of research must be considered. By embracing multiple areas of research, practitioners and policymakers can better integrate knowledge from disparate areas of research, such as urban planning, landscape architecture, sociology, governance, public health, leisure and recreational studies, and psychology to encourage green space use and to deliver benefits. Although we discuss these areas of research broadly in the present article, we acknowledge that there are other areas of research that have not been included but that should be brought into the ongoing discussion of human–environment interactions and benefits to society. The following section addresses primarily planning, in recognition that these more top-down approaches need to be complemented and sometimes informed by other actor initiatives, bottom-up or emerging from different actor coalitions. For example, there are now many examples of community led initiatives and alternative governance arrangements (Buijs et al. 2016).

Local governments acknowledge that urban residents are not homogenous in their values and desires for green spaces and may differ in their perceptions and preferences for the same space. Discerning how green space attributes match individual and community needs is a complex formulation that still requires both theoretical and practical research to understand how to design green spaces across an urban landscape. A wide range of stakeholders necessitates a wide range of services and functions to truly serve a diverse public, especially when considering multicultural communities and changing requirements of individuals and communities through time (Neal et al. 2015, Douglas et al. 2017).

Developing policies that provide a pathway for people to use green space first requires a clearer appreciation of the relationship between people and green space environments (Ives et al. 2017). Gaining a clearer understanding of how and why individuals differ in their values and decision-making about park use can help policymakers uncover simple levers that are needed or already available to increase green space visitation. The two concepts presented in the present article, affordances and institutional processes, provide a bridge for integrating disciplinary information through a relational understanding of the human–environment interaction (affordance) while also providing an active process for changing and adjusting these relationships (institutional or social processes). Affordances allow for the complexity of an individual's relationship to change through space, time, and circumstance as both the person and the environment around the person change. These changes may be positive or negative in regards to the relationship between an individual and a specific location, because the individual may see their environment as providing more or less of what they desire. However, institutional and social processes play a role in providing a lever for adjusting these relationships through active work with communities to consider novel and potentially more beneficial ways for them to consider and interact with their environment. For example, programs that teach a community about the ecology of a wetland may change the perception of that wetland from a putrid, mosquito-producing swamp to an area of bird watching and where water is naturally filtered.

Practical processes must also be developed and implemented in order to test these theories. Solutions such as community consultation and focus groups have been used as a way to elicit viewpoints and information from specific segments of the population in park design projects (Krueger 2014, Yung et al. 2016, Ryan et al. 2020). The codesign of public spaces with local communities to provide an interdisciplinary public participation activity have also been explored in many cities around the world to ensure that the political, social, and economic priorities are based on a broad consensus of decision-making and that the most excluded, poorest, or most vulnerable voices are also heard (Clever Cities 2021, Remesar 2021). In an Australian example, Brimbank Council purchased land to increase the open space availability in an area lacking public space. The community submitted 548 codesigned ideas and worked with the council to advocate to embed aspects of an old school site, to provide facilities that were popular with refugees within the area, and to incorporate art of the local Indigenous groups (AIPH 2022).

Such institutional processes require political will and resources to implement but also require the correct facilitation to ensure that power imbalances do not threaten the viability of the process (Nyumba et al. 2018, Bogle et al. 2019). The governance of public spaces can be contentious, depending on the stakeholder and the conflicting demands across user groups, and often, planning does not provide space and time for community dialogue and discussion to occur before the green space design is completed (Lindholst et al. 2016). Such spaces for discussion and consideration are increasingly valued, because they break down siloes and strengthen social networks (Kronenberg et al. 2016). Although the current processes of community consultation may not always account for differing perspectives appropriately, clearly thought-out designs will help move processes forward in a way that allows for greater inclusion in discussions and that will take into account the particular context of each situation.

Although parks are designed to be multifunctional, infrastructure and landscapes structures are often hardwired in place, leaving little room for flexibility in design and use under changing circumstances. Leaving room in the design that allows spaces to be flexible may be important in future circumstances as new needs emerge and in potentially sudden and unexpected ways. These new design needs may emerge because of novel situations that affect the community, such as what we saw with the COVID-19 pandemic, during which people wanted to be in green spaces but in a socially distanced manner (Venter et al. 2020, Lopez et al. 2021) and where individuals with little or no private green space relied more heavily on public green spaces to spend time outdoors (Poortinga et al. 2021). A changing climate may also create challenging requirements of urban green space, such as communities needing to spend time in cool green spaces during extreme heatwaves, but drought conditions may prevent the irrigation of shade trees or water features that provide cooling (Brown et al. 2015, Doll et al. 2022). This would require planning on how to accommodate greater use of the park while providing the cooling services desired under climatically difficult circumstances. The community may also change around already established green spaces, as has been seen through time in cities because of domestic and international migration, as well as gentrification, and different desires of park use and signals of place making may be required (Curran and Hamilton 2017, Burns and Berbary 2021). However, programming and institutional processes may also be required to help new communities engage with local green spaces. With a changing context, environment, and community through time, active management of the landscape, as well as programming to meet the needs of individuals of the city, may be required so that affordances can adjust to new circumstances and so that benefits can continue to be delivered.

To truly bring about the benefits of green spaces for communities, we will have to actively develop plural modes of engagement and learning, processes that encourage individuals to imagine and develop agency within green spaces. Such processes will require time and resources that are limited but have potential value over an individual's lifetime.

## Author Biographical

Brenda B. Lin (brenda.lin@csiro.au) is affiliated with CSIRO Land and Water, in Brisbane, Queensland, Australia. Erik Andersson is affiliated with the Stockholm Resilience Centre, at Stockholm University, in Stockholm, Sweden; with the Ecosystems and Environment Research Program at the University of Helsinki, in Helsinki, Finland; and with the Research Unit for Environmental Sciences and Management at North-West University, in Vanderbijlpark, South Africa.
